# 4-Nitro­benzyl 2-chloro­acetate

**DOI:** 10.1107/S1600536809019278

**Published:** 2009-05-29

**Authors:** Kai Zhu, Yan-Hua Wang, Hui Liu, Ping-Fang Han, Ping Wei

**Affiliations:** a College of Biotechnology and Pharmaceutical Engineering, Nanjing University of Technology, Xinmofan Road No. 5 Nanjing, Nanjing 210009, People’s Republic of China

## Abstract

In the mol­ecule of the title compound, C_9_H_8_ClNO_4_, the nearly planar acetate moiety [maximum deviation = 0.015 (3) Å for an O atom] is oriented with respect to the plane of the aromatic ring at a dihedral angle of 73.03 (3)°. In the crystal structure, inter­molecular C—H⋯O inter­actions link mol­ecules into a network. π–π contacts between benzene rings [centroid–centroid distance = 4.000 (1) Å] may further stabilize the structure.

## Related literature

For a related structure, see: Pyun *et al.* (2001 ▶). For bond-length data, see: Allen *et al.* (1987 ▶).
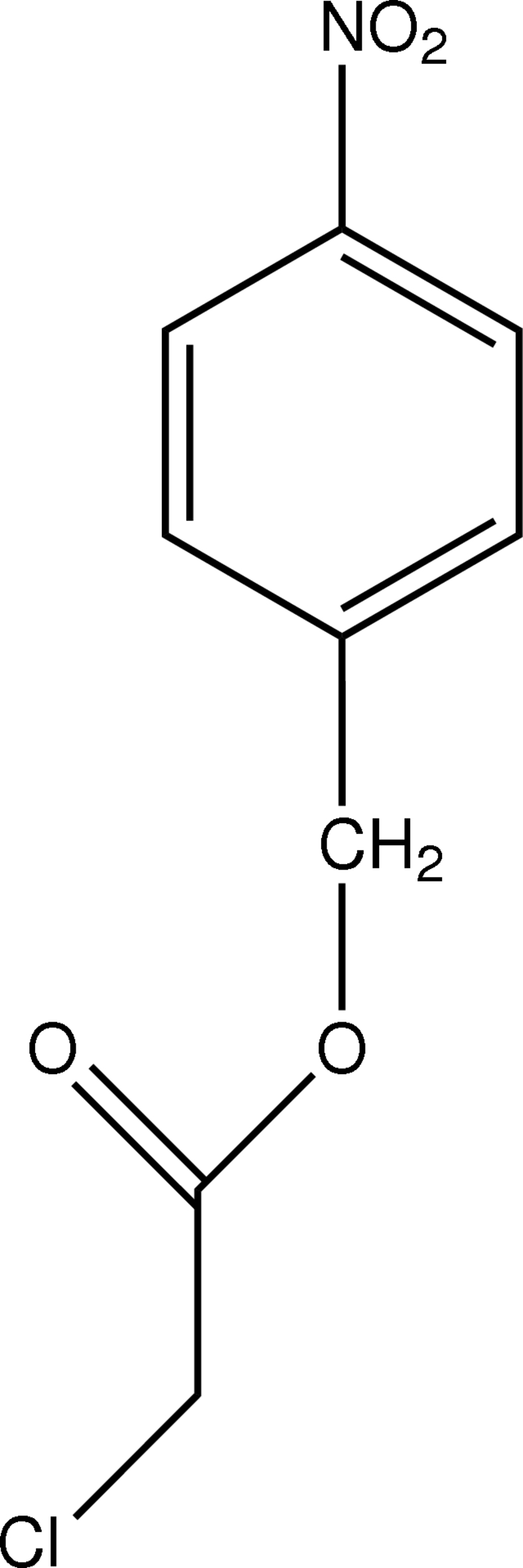


## Experimental

### 

#### Crystal data

C_9_H_8_ClNO_4_
*M* *_r_* = 229.61
Monoclinic, 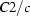

*a* = 13.636 (3) Å
*b* = 8.1570 (16) Å
*c* = 18.878 (4) Å
β = 108.30 (3)°
*V* = 1993.5 (8) Å^3^
*Z* = 8
Mo *K*α radiation
μ = 0.38 mm^−1^
*T* = 294 K
0.30 × 0.20 × 0.10 mm

#### Data collection

Enraf–Nonius CAD-4 diffractometer
Absorption correction: ψ scan (North *et al.*, 1968 ▶) *T*_min_ = 0.896, *T*_max_ = 0.963
1892 measured reflections
1814 independent reflections
1132 reflections with *I* > 2σ(*I*)
*R*_int_ = 0.055
3 standard reflections frequency: 120 min intensity decay: 1%

#### Refinement

*R*[*F* ^2^ > 2σ(*F* ^2^)] = 0.068
*wR*(*F* ^2^) = 0.191
*S* = 1.00
1814 reflections
136 parameters
H-atom parameters constrained
Δρ_max_ = 0.31 e Å^−3^
Δρ_min_ = −0.28 e Å^−3^

### 

Data collection: *CAD-4 Software* (Enraf–Nonius, 1989 ▶); cell refinement: *CAD-4 Software*; data reduction: *XCAD4* (Harms & Wocadlo, 1995 ▶); program(s) used to solve structure: *SHELXS97* (Sheldrick, 2008 ▶); program(s) used to refine structure: *SHELXL97* (Sheldrick, 2008 ▶); molecular graphics: *ORTEP-3 for Windows* (Farrugia, 1997 ▶); software used to prepare material for publication: *SHELXL97* and *PLATON* (Spek, 2009 ▶).

## Supplementary Material

Crystal structure: contains datablocks global, I. DOI: 10.1107/S1600536809019278/hk2695sup1.cif

Structure factors: contains datablocks I. DOI: 10.1107/S1600536809019278/hk2695Isup2.hkl

Additional supplementary materials:  crystallographic information; 3D view; checkCIF report

## Figures and Tables

**Table 1:** Hydrogen-bond geometry (Å, °)

*D*—H⋯*A*	*D*—H	H⋯*A*	*D*⋯*A*	*D*—H⋯*A*
C1—H1*B*⋯O1^i^	0.97	2.35	3.275 (5)	160
C3—H3*A*⋯O1^ii^	0.97	2.58	3.456 (5)	151

Symmetry codes: (i) 
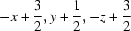
; (ii) 
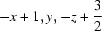
.

**Fig. 1.**
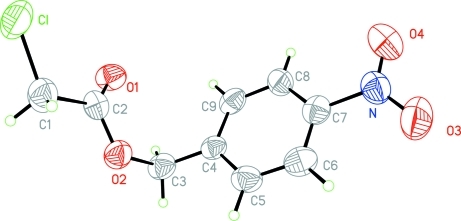
The molecular structure of the title molecule, with the atom-numbering scheme. Displacement ellipsoids are drawn at the 30% probability level.

**Fig. 2.**
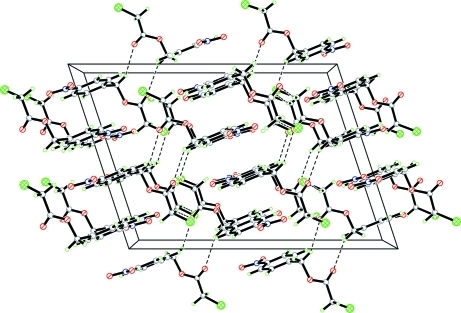
A partial packing diagram of the title compound. Hydrogen bonds are shown as dashed lines.

## supplementary crystallographic information

### Comment

Some derivatives of *p*-nitrobenzyl alcohol are important chemical materials. We report herein the crystal structure of the title compound.

In the molecule of the title compound (Fig 1), the bond lengths (Allen *et al.*,  1987) and angles are within normal ranges. Ring A (C4-C9) is, of course, planar. Atoms N, C3, O3 and O4 are 0.005 (3), 0.027 (3), -0.146 (3) and 0.144 (4) Å away from the ring plane, respectively. On the other hand, (O1/O2/C1-C3) moiety is nearly planar with a maximum deviation of 0.015 (3) Å for atom O2 and it is oriented with respect to ring A at a dihedral angle of 73.03 (3)°.

In the crystal structure, intermolecular C-H···O interactions (Table 1) link the molecules into a network (Fig. 2), in which they may be effective in the stabilization of the structure. The π–π contact between the benzene rings, Cg1—Cg1^i^ [symmetry code: (i) 1 - x, -y, -z, where Cg1 is centroid of the ring A (C4-C9) may further stabilize the structure, with centroid-centroid distance of 4.000 (1) Å.

### Experimental

For the preparation of the title compound, chloroacetyl chloride (1.1 g) and *p*-nitrobenzyl alcohol (1.53 g) were added into the mixture of pyridine (15 ml) and dichloromethane (30 ml) at 273–278 K. The gross products were extracted with n-hexane, washed with water, and dried under vaccum, and then recrystallized in dichloromethane (yield; 0.916 g) (Pyun *et al.*, 2001). Crystals suitable for X-ray analysis were obtained by slow evaporation of a methanol solution.

### Refinement

H atoms were positioned geometrically, with C-H = 0.93 and 0.97 Å for aromatic and methylene H, respectively, and constrained to ride on their parent atoms, with U_iso_(H) = 1.2U_eq_(C).

### Crystal data

**Table:**

C_9_H_8_ClNO_4_	*F*(000) = 944
*M**_r_* = 229.61	*D*_x_ = 1.530 Mg m^−^^3^
Monoclinic, *C*2/*c*	Mo *K*α radiation, λ = 0.71073 Å
Hall symbol: -C 2yc	Cell parameters from 25 reflections
*a* = 13.636 (3) Å	θ = 9–13°
*b* = 8.1570 (16) Å	µ = 0.38 mm^−^^1^
*c* = 18.878 (4) Å	*T* = 294 K
β = 108.30 (3)°	Block, colorless
*V* = 1993.5 (8) Å^3^	0.30 × 0.20 × 0.10 mm
*Z* = 8	

### Data collection

**Table:**

Enraf–Nonius CAD-4 diffractometer	1132 reflections with *I* > 2σ(*I*)
Radiation source: fine-focus sealed tube	*R*_int_ = 0.055
graphite	θ_max_ = 25.3°, θ_min_ = 2.3°
ω/2θ scans	*h* = −16→0
Absorption correction: ψ scan (North *et al.*, 1968)	*k* = −9→0
*T*_min_ = 0.896, *T*_max_ = 0.963	*l* = −21→22
1892 measured reflections	3 standard reflections every 120 min
1814 independent reflections	intensity decay: 1%

### Refinement

**Table:**

Refinement on *F*^2^	Primary atom site location: structure-invariant direct methods
Least-squares matrix: full	Secondary atom site location: difference Fourier map
*R*[*F*^2^ > 2σ(*F*^2^)] = 0.068	Hydrogen site location: inferred from neighbouring sites
*wR*(*F*^2^) = 0.191	H-atom parameters constrained
*S* = 1.00	*w* = 1/[σ^2^(*F*_o_^2^) + (0.1*P*)^2^ + 2*P*] where *P* = (*F*_o_^2^ + 2*F*_c_^2^)/3
1814 reflections	(Δ/σ)_max_ < 0.001
136 parameters	Δρ_max_ = 0.31 e Å^−^^3^
0 restraints	Δρ_min_ = −0.28 e Å^−^^3^

### Special details

**Table:**

Geometry. All e.s.d.'s (except the e.s.d. in the dihedral angle between two l.s. planes) are estimated using the full covariance matrix. The cell e.s.d.'s are taken into account individually in the estimation of e.s.d.'s in distances, angles and torsion angles; correlations between e.s.d.'s in cell parameters are only used when they are defined by crystal symmetry. An approximate (isotropic) treatment of cell e.s.d.'s is used for estimating e.s.d.'s involving l.s. planes.
Refinement. Refinement of *F*^2^ against ALL reflections. The weighted *R*-factor *wR* and goodness of fit *S* are based on *F*^2^, conventional *R*-factors *R* are based on *F*, with *F* set to zero for negative *F*^2^. The threshold expression of *F*^2^ > σ(*F*^2^) is used only for calculating *R*-factors(gt) *etc*. and is not relevant to the choice of reflections for refinement. *R*-factors based on *F*^2^ are statistically about twice as large as those based on *F*, and *R*- factors based on ALL data will be even larger.

### Fractional atomic coordinates and isotropic or equivalent isotropic displacement parameters (Å^2^)

**Table:**

	*x*	*y*	*z*	*U*_iso_*/*U*_eq_	
Cl	0.85639 (10)	0.10201 (17)	0.74171 (7)	0.0745 (5)	
O1	0.6449 (2)	0.0812 (4)	0.75755 (16)	0.0561 (8)	
O2	0.6848 (2)	0.2403 (4)	0.86053 (15)	0.0528 (8)	
O3	0.6555 (4)	−0.3688 (5)	1.1134 (2)	0.1110 (17)	
O4	0.6159 (3)	−0.5146 (4)	1.0154 (2)	0.0854 (11)	
N	0.6314 (3)	−0.3823 (5)	1.0467 (2)	0.0567 (10)	
C1	0.8107 (3)	0.2211 (5)	0.8028 (3)	0.0578 (11)	
H1A	0.8582	0.2114	0.8531	0.069*	
H1B	0.8085	0.3355	0.7883	0.069*	
C2	0.7037 (3)	0.1679 (5)	0.8022 (2)	0.0444 (9)	
C3	0.5855 (3)	0.2023 (5)	0.8709 (2)	0.0531 (11)	
H3A	0.5334	0.1868	0.8228	0.064*	
H3B	0.5641	0.2927	0.8961	0.064*	
C4	0.5956 (3)	0.0483 (5)	0.9169 (2)	0.0411 (9)	
C5	0.6365 (3)	0.0540 (5)	0.9945 (2)	0.0518 (10)	
H5A	0.6568	0.1545	1.0177	0.062*	
C6	0.6476 (3)	−0.0835 (5)	1.0369 (2)	0.0544 (11)	
H6A	0.6741	−0.0768	1.0886	0.065*	
C7	0.6192 (3)	−0.2331 (5)	1.0024 (2)	0.0465 (10)	
C8	0.5789 (3)	−0.2437 (5)	0.9254 (2)	0.0505 (10)	
H8A	0.5596	−0.3446	0.9023	0.061*	
C9	0.5679 (3)	−0.1045 (5)	0.8843 (2)	0.0507 (10)	
H9A	0.5411	−0.1116	0.8326	0.061*	

### Atomic displacement parameters (Å^2^)

**Table:**

	*U*^11^	*U*^22^	*U*^33^	*U*^12^	*U*^13^	*U*^23^
Cl	0.0709 (9)	0.0894 (9)	0.0776 (9)	−0.0099 (7)	0.0438 (7)	−0.0052 (7)
O1	0.0460 (17)	0.0621 (18)	0.0581 (17)	−0.0091 (14)	0.0132 (14)	−0.0194 (15)
O2	0.0569 (18)	0.0581 (17)	0.0450 (15)	−0.0098 (14)	0.0182 (13)	−0.0066 (13)
O3	0.195 (5)	0.079 (3)	0.056 (2)	−0.008 (3)	0.036 (3)	0.008 (2)
O4	0.104 (3)	0.056 (2)	0.097 (3)	0.005 (2)	0.033 (2)	0.009 (2)
N	0.055 (2)	0.052 (2)	0.066 (3)	0.0018 (18)	0.0231 (19)	0.0086 (19)
C1	0.054 (3)	0.061 (3)	0.060 (3)	−0.012 (2)	0.020 (2)	−0.004 (2)
C2	0.040 (2)	0.043 (2)	0.046 (2)	−0.0012 (19)	0.0078 (18)	0.0066 (19)
C3	0.045 (2)	0.059 (3)	0.059 (3)	0.0026 (19)	0.023 (2)	−0.004 (2)
C4	0.036 (2)	0.047 (2)	0.044 (2)	−0.0013 (17)	0.0165 (16)	−0.0054 (17)
C5	0.053 (3)	0.048 (2)	0.055 (3)	−0.007 (2)	0.018 (2)	−0.012 (2)
C6	0.052 (3)	0.061 (3)	0.046 (2)	−0.003 (2)	0.0091 (19)	−0.010 (2)
C7	0.035 (2)	0.056 (2)	0.051 (2)	0.0016 (19)	0.0182 (18)	0.002 (2)
C8	0.056 (3)	0.044 (2)	0.052 (2)	−0.011 (2)	0.019 (2)	−0.004 (2)
C9	0.051 (2)	0.063 (3)	0.039 (2)	−0.008 (2)	0.0163 (18)	−0.009 (2)

### Geometric parameters (Å, °)

**Table:**

Cl—C1	1.765 (4)	C3—H3B	0.9700
O1—C2	1.195 (5)	C4—C9	1.389 (5)
O2—C2	1.344 (5)	C4—C5	1.394 (5)
O2—C3	1.461 (5)	C5—C6	1.359 (6)
N—O3	1.203 (5)	C5—H5A	0.9300
N—O4	1.217 (5)	C6—C7	1.381 (6)
N—C7	1.457 (5)	C6—H6A	0.9300
C1—C2	1.519 (6)	C7—C8	1.385 (6)
C1—H1A	0.9700	C8—C9	1.357 (6)
C1—H1B	0.9700	C8—H8A	0.9300
C3—C4	1.509 (6)	C9—H9A	0.9300
C3—H3A	0.9700		
			
C2—O2—C3	116.2 (3)	C9—C4—C5	117.4 (4)
O3—N—O4	122.7 (4)	C9—C4—C3	121.9 (3)
O3—N—C7	118.0 (4)	C5—C4—C3	120.7 (4)
O4—N—C7	119.4 (4)	C6—C5—C4	121.7 (4)
C2—C1—Cl	111.9 (3)	C6—C5—H5A	119.2
C2—C1—H1A	109.2	C4—C5—H5A	119.2
Cl—C1—H1A	109.2	C5—C6—C7	119.3 (4)
C2—C1—H1B	109.2	C5—C6—H6A	120.4
Cl—C1—H1B	109.2	C7—C6—H6A	120.4
H1A—C1—H1B	107.9	C6—C7—C8	120.7 (4)
O1—C2—O2	125.3 (4)	C6—C7—N	120.2 (4)
O1—C2—C1	127.2 (4)	C8—C7—N	119.1 (4)
O2—C2—C1	107.4 (3)	C9—C8—C7	118.9 (4)
O2—C3—C4	109.5 (3)	C9—C8—H8A	120.5
O2—C3—H3A	109.8	C7—C8—H8A	120.5
C4—C3—H3A	109.8	C8—C9—C4	122.1 (4)
O2—C3—H3B	109.8	C8—C9—H9A	119.0
C4—C3—H3B	109.8	C4—C9—H9A	119.0
H3A—C3—H3B	108.2		
			
C3—O2—C2—O1	2.8 (6)	C5—C6—C7—N	179.3 (4)
C3—O2—C2—C1	−178.9 (3)	O3—N—C7—C6	8.3 (6)
Cl—C1—C2—O1	−14.4 (6)	O4—N—C7—C6	−172.6 (4)
Cl—C1—C2—O2	167.3 (3)	O3—N—C7—C8	−171.9 (5)
C2—O2—C3—C4	86.8 (4)	O4—N—C7—C8	7.3 (6)
O2—C3—C4—C9	−96.3 (4)	C6—C7—C8—C9	0.1 (6)
O2—C3—C4—C5	81.6 (5)	N—C7—C8—C9	−179.7 (4)
C9—C4—C5—C6	−1.1 (6)	C7—C8—C9—C4	−0.2 (6)
C3—C4—C5—C6	−179.1 (4)	C5—C4—C9—C8	0.7 (6)
C4—C5—C6—C7	1.0 (7)	C3—C4—C9—C8	178.6 (4)
C5—C6—C7—C8	−0.5 (6)		

### Hydrogen-bond geometry (Å, °)

**Table:**

*D*—H···*A*	*D*—H	H···*A*	*D*···*A*	*D*—H···*A*
C1—H1B···O1^i^	0.97	2.35	3.275 (5)	160
C3—H3A···O1^ii^	0.97	2.58	3.456 (5)	151

Symmetry codes: (i) −*x*+3/2, *y*+1/2, −*z*+3/2; (ii) −*x*+1, *y*, −*z*+3/2.
